# Developmental Immunotoxicity, Perinatal Programming, and Noncommunicable Diseases: Focus on Human Studies

**DOI:** 10.1155/2014/867805

**Published:** 2014-01-23

**Authors:** Rodney R. Dietert

**Affiliations:** Department of Microbiology and Immunology, College of Veterinary Medicine, Cornell University, North Tower Road, Ithaca, NY 14853, USA

## Abstract

Developmental immunotoxicity (DIT) is a term given to encompass the environmentally induced disruption of normal immune development resulting in adverse outcomes. A myriad of chemical, physical, and psychological factors can all contribute to DIT. As a core component of the developmental origins of adult disease, DIT is interlinked with three important concepts surrounding health risks across a lifetime: (1) the Barker Hypothesis, which connects prenatal development to later-life diseases, (2) the hygiene hypothesis, which connects newborns and infants to risk of later-life diseases and, (3) fetal programming and epigenetic alterations, which may exert effects both in later life and across future generations. This review of DIT considers: (1) the history and context of DIT research, (2) the fundamental features of DIT, (3) the emerging role of DIT in risk of noncommunicable diseases (NCDs) and (4) the range of risk factors that have been investigated through human research. The emphasis on the human DIT-related literature is significant since most prior reviews of DIT have largely focused on animal research and considerations of specific categories of risk factors (e.g., heavy metals). Risk factors considered in this review include air pollution, aluminum, antibiotics, arsenic, bisphenol A, ethanol, lead (Pb), maternal smoking and environmental tobacco smoke, paracetamol (acetaminophen), pesticides, polychlorinated biphenyls, and polyfluorinated compounds.

## 1. Introduction

Early-life environmental insults affecting the developing immune system can have significant health ramifications not only for the exposed offspring but also potentially extending to additional generations. Developmental immunotoxicity (DIT) appears to play a significant role in the current global epidemic of non-communicable diseases (NCDs) [1, 2]. This review of DIT begins with the history of DIT placed in the context of the area of immunology known as immunotoxicology and charts the emergence of recent concepts concerning early developmental programming as it impacts later-life health. It also describes the current state of the science for DIT and the likely applications of DIT assessment as it may impact both human health and environmental protection. In particular, the paper discusses (1) the history of DIT research, (2) the role of critical windows of vulnerability for the developing immune system, (3) frequent outcomes of DIT, (4) consideration of the microbiome in DIT, (5) the role of prenatal epigenetic alterations in immunotoxicity, and (6) the connection between DIT, elevated risk of comorbid chronic diseases, and current epidemic of NCDs.

## 2. History of DIT Research

### 2.1. Emergence within Immunotoxicology

Immunotoxicology, the study of the adverse impact of environmental conditions (e.g., exposure to food, drugs, chemicals, microbial agents, and physical and psychosocial factors) on the immune system, began to gain recognition during the 1970s and early 1980s [3] with the initial focus on use of surrogates for host resistance in animal models [4, 5] and concern about environmentally induced immunosuppression [6]. The search for assays and parameters that were predictive of chemical- or drug-induced immunotoxicity centered on measures that could replace then cumbersome and costly host challenges with infectious agents or transplantable cancer cells. Not surprisingly loss of immune protection (i.e., immunosuppression) and increased susceptibility to infections and/or cancer were a driving concern. This also coincided with the era in which the HIV-induced immunosuppression associated with AIDS was an increasing human health challenge [7, 8]. A coordinated effort to identify the best predictor of immunotoxicity resulted in the development of the tier system of assays providing a strategy of immunotoxicity testing [9] and the concept that a limited combination of immune measures could be effective for identifying immunotoxic chemicals [10, 11]. In addition to the identification of chemicals that could produce immunosuppression in humans [12], the detection of chemicals with sensitizing potential was an early systematic concern within immunotoxicology [13, 14].

The examination of adverse insults to the developing immune system, a subsection of immunotoxicology known as developmental immunotoxicity (DIT), was among the first research initiatives within immunotoxicology. As early as the 1970s, animal studies revealed the persistent nature of immune problems resulting from early-life insult. Studies involving drugs [15, 16], heavy metals [17, 18], pesticides [19], mold toxins [20–22], and polycyclic aromatic hydrocarbons [23] suggested that the developmental periods of immune system formation, dissemination, and acquired host defense capacities represent developmental windows that need to be research and public safety priorities. Yet, developmental studies represented only one of several aspects of immunotoxicological research and the term “developmental immunotoxicity” was not prevalent in the literature until the mid-1990s [24, 25].

DIT did not achieve a priority position for research and safety evaluation within immunotoxicology until approximately the late 1990s–early 2000s. Among the important events were a workshop on childhood health risks coordinated by the March of Dimes and EPA [26–28], the publication of a seminar text on compiling DIT research [29], and the increasing recognition of fetal programming of later-life health and disease [30–32]. Basic features of developmental immunotoxicity (DIT) have emerged during decades of research. These features are shown as follow

DIT

- is directly linked with immune dysfunction and increased risk of NCDs,
- stems from critical developmental windows of immune vulnerability restricted to the young,
- can happen at lower exposure levels than usually produce adult-exposure immunotoxicity,
- often involves a broader spectrum of adverse immune outcome versus adult-exposure immunotoxicity,
- usually produces more persistent effects than those following adult exposure,
- can lead to latent dysfunction that may be masked until it is triggered by a later-life event,
- often manifests as immune dysfunctional imbalances (suppression of some immune responses along with the inappropriate enhancement of others),
- may produce different sex-based outcomes,
- is not routinely assessed in most required safety testing protocols to date,
- can occur via several different biological pathways (e.g., impaired immune maturation, epigenetic alteration, and immune-microbiome disruption).

The DIT literature is sufficiently extensive to permit fundamental characterizations. This information is derived from [1, 27, 33–40].

### 2.2. DIT and the Barker Hypothesis

The impetus for a greater focus on DIT was aided by the findings of Barker and colleagues that maternal undernutrition during prenatal development could increase the risk of cardiovascular disease (CVD) in the offspring [41–43]. This led to what has been termed the “Barker Hypothesis” [44]. Originally, the linkage between fetal environment-adult disease was focused solely on maternal nutrition and CVD (including both coronary heart disease and hypertension) as an example linking early developmental conditions and fetal programming to later-life adult disease. But it became clear that the same relationship could exist for many other adult chronic diseases and conditions (e.g., renal disease and type 2 diabetes, in adult offspring that were also affected by the fetal nutritional environment) [45, 46].

### 2.3. DIT and Developmental Origins of Adult Health and Disease (DOHaD)

As the net was cast beyond just maternal-fetal nutritional status to include a wide array of environmental conditions and factors, the concept of developmental origins of health and disease (DOHaD) emerged [47, 48] to connect critical windows of development with specific childhood and adult health risks. Immune damage, dysfunction, and/or imbalances are now known to persist long after either toxicant levels of chemical exposures return to normal or physical-psychosocial stressors have been removed [33, 34]. In fact, part of the challenge in deciphering pathways resulting in DIT and fetal programming of adverse immune status is that evidence of prior problematic exposure conditions may remain largely hidden. For this reason, DIT testing usually requires careful consideration about exposure windows and immunological assessment tools [35, 36, 49]. The opportunity to examine the different functional responses of the immune system in response to various challenges has emerged as a key component of safety assessment [35, 50, 51].

## 3. Fundamental Features of DIT

### 3.1. Heightened Sensitivity of the Developing Immune System

One of the hallmarks of the developing immune system is that it exhibits an increased sensitivity for most environmentally induced toxicity compared with the fully matured immune system of the adult. Additionally, DIT often occurs at exposure doses that are below those producing other developmental effects [52–55]. Luebke et al. [33] reviewed the evidence of comparative age-based sensitivity for five of the most extensively studied drugs and environmental chemicals: diethylstilbestrol (DES), diazepam (DZP), lead (Pb), 2,3,7,8-tetrachlorodibenzo-p-dioxin (TCDD), and tributyltin oxide (TBO). They concluded that early development appears to be a time of increased sensitivity to xenobiotics and risk of adverse immune outcomes that are likely to persist into later life.

This increased risk of developmental immune insult compared with that of the adult has been seen across broad categories of drugs and chemicals as well as among different dietary and physical/psychological factors [1, 29, 37]. This differential, age-based sensitivity can take different forms, which are reviewed in detail in Dietert and Piepenbrink [38]. In many cases, the lowest dose required to produce immune disruption is several fold to several magnitudes lower in early life than in the adult [33]. Additionally, a broader array of immune parameters are likely to be affected following exposure of the nonadult versus the adult [54, 56]. Prenatal and early postnatal exposures are more likely to produce persistent adverse immune outcomes [57–60].

### 3.2. Critical Developmental Windows

The identification and consideration of both systemic and tissue-oriented developmental vulnerabilities for the immune system have undergone progressive evolution since the original series of immune “critical windows” emerged from a national workshop [27, 28]. As was illustrated in Dietert [1], for most key developmental steps of immune maturation, multiple environmental disruptors have been identified. The effect of inhibition or delay of a critical developmental step can increase the risk of multiple later-life diseases. For example, key processes of T cell selection in the thymus can be affected by maternal exposure to certain heavy metals, plasticizers, dioxins, polycyclic chlorinated biphenyls, tobacco smoke, and certain drugs. Not surprisingly, the adverse health outcomes that have been associated with environmental targeting of thymus-directed processes are largely restricted to prenatal development and cover virtually every category of disease including cancer as well as autoimmune and allergic diseases and childhood vaccine failures [1].

Each immune developmental window has its own unique vulnerabilities that are best detected via age-relevant safety screening [38]. For example, Bunn et al. [61] demonstrated that while Pb was immunotoxic across all windows of prenatal developmental, later gestational maternal exposures were more likely to result in profound T helper 2- (Th2-) favored functional skewing in the juvenile rat.

Application of the critical windows concept for enhanced immune-associated disease prevention has been explored by Jenmalm and Duchén [62]. These authors stressed that dietary interventions capable of aiding prevention of allergy are most likely to be effective if directed toward specific prenatal, perinatal, and early postnatal developmental windows [62].

## 4. Frequent Outcomes of DIT and Risk of Noncommunicable Diseases (NCDs)

One of the outcomes of the recent human studies on DIT and fetal programming of immune-based disease is an increasing realization that these processes are major contributors to the ongoing epidemic of noncommunicable diseases (NCDs) (most of which are chronic diseases). NCDs are the major cause of death globally and include cardiovascular disease as well as cancer [63]. What has become clear is that the vast majority of NCDs cannot be maintained in the absence of misregulated (usually unresolving) inflammation [39, 64, 65]. This means that improper immune homeostasis in tissues is likely to be required for NCD onset and/or maintenance. Human studies supported by animal model research have established the importance of the prenatal and early postnatal environment for maturation of innate immune cells in concert with formation of the microbiome in mucosal tissues and other sites (e.g., skin).

One of the impediments to recognizing this DIT-NCD linkage is that historic examination of immunotoxicity often focused on changes in primary and secondary immune organs. However, the majority of immune cells actually reside outside of these organs in mucosal and other tissues such as the gut, brain, skin, liver, endocrine, reproductive, urogenital, and cardiovascular systems. It is these tissues that are most often involved with NCDs, and the status of these resident immune cells is often at issue relative to tissue pathology. A shift in focus in immune evaluation to consider the impact of DIT and later-life status of cells such as skin dendritic cells, microglia, Kupffer cells, and immune cells of the BALT and GALT should provide a clearer picture of the cause-effect relationship between DIT and certain NCDs [66].  Table 1 provides examples of environmental factors and conditions that are thought to contribute to later-life human disease via DIT and immune dysfunction. NCDs represent the majority of examples shown in Table 1.

The significance of the prevention of DIT as a strategy to reduce the prevalence of NCDs has been strengthened with the awareness that NCDs exist as tightly intertwined patterns of comorbid risks. This paradigm of tightly interlinked chronic diseases and conditions was described in a series of papers illustrating the health risk trajectories that exist when children are diagnosed with a number of different immune-/inflammatory-driven conditions: asthma, recurrent infections, schizophrenia, autoimmune thyroiditis, celiac disease, inflammatory disease, and psoriasis [2, 34, 67, 68]. Cancer is one of the common outcomes in the tissue receiving the primary inflammatory insult [40]. Even end-stage conditions such as chronic kidney disease and frailty form part of these interlinked patterns of chronic diseases and conditions [39, 69].

As an example, one interconnected pattern of comorbidity exists among a triad of autoimmune conditions: type 1 diabetes, celiac disease, and autoimmune thyroiditis. Children diagnosed with type 1 diabetes have a predictably greater risk for developing celiac disease and/or autoimmune thyroiditis [70, 71]. While the mechanism remains to be elucidated, childhood asthma, obesity, and sleep disorders are similarly interlinked in a triad [72, 73]. Tanaka et al. [74] and Anders et al. [75] have pointed out that clinical depression is a largely immune driven, inflammatory-based condition that is another comorbid outcome intrinsically connected to many different NCDs/chronic diseases.

The ramification of these comorbid disease interconnections is that there is increased value in avoiding fetal programming that results in childhood-onset, immune dysfunction-based NCDs. These implications led four immunotoxicologists to call for required DIT testing of chemicals and drugs as a step to better protect children from the risk of NCDs [2].

## 5. Human Studies Involving DIT: Alphabetical List of Risk Factors

Most prior reviews of DIT have focused largely on animal research. This section examines the wide range of risk factors for DIT that has been evaluated among human populations. Evidence supporting the occurrence of DIT among human populations has been obtained from both exposed populations as well as via epidemiological studies. The risk factors are presented alphabetically rather than being grouped into different categories (e.g., chemicals, drugs, physical, and psychological factors).

In many of these studies antibody titers against either a common virus or childhood vaccinations have been used as a biomarker of DIT. While limited as an overall immune measure, there are significant benefits to this approach: (1) serum antibody levels are easily determined, (2) a majority of children will have been vaccinated according to a predictable and standard schedule, and (3) the microbial infection or vaccine challenge of the child's immune system will enable a detection of potential dysfunction in an actively responding immune system and, based on animal data, these are among the most sensitive parameters for measuring DIT. Other studies reach beyond vaccination data to examine associations between exposure/environmental conditions and immune-based chronic diseases during childhood. Among the most commonly used are asthma, allergic rhinitis, atopic dermatitis, type 1 diabetes, celiac disease, and inflammatory bowel disease. Only a portion of these disease-association studies has overt human immune function associated with them. For the remainder, there has been a tendency to rely more on linking DIT immune function animal data with information on human immune disease-associations.

### 5.1. Air Pollution

Ambient air pollution including specific components (e.g., polycyclic aromatic hydrocarbons, particulate matter) has been implicated in respiratory and cardiovascular diseases via improperly controlled inflammation. Nadeau et al. [76] examined groups of asthmatic and nonasthmatic children in Fresno, CA, for pollutant exposure, T regulatory (Treg) cell activity (that would help to control Th2 mediated asthma symptoms), and DNA methylation. The researchers found that increased exposure to ambient air pollutants was associated with increased methylation of CpG islands at the Foxp3 locus as well as reduced Foxp3 expression [76]. They also reported reduced numbers of Fox3p+ Treg cells and reduced Treg activity particularly among the asthmatic children. The authors concluded that increased air pollution exposure in children is associated with increased asthma morbidity via epigenetic alterations and a possible immune mechanism [76].

Kerkhof et al. [77] found evidence in children that traffic-related air pollution (e.g., particulate matter (PM) 2.5, 10, soot, and nitrogen dioxide) increased the prevalence of doctor-diagnosed asthma by year 8 particularly among children with specific variant alleles for the toll-like receptor (TLR) genes 2 and 4. The investigators suggested that their results are consistent with the suspected involvement of innate immune response in the linkage between exposure to traffic pollution and risk of childhood asthma [77].

Calderón-Garcidueñas et al. [78] compared immune markers in asymptomatic children from two different city areas (Southwest Mexico City versus Polotitlán, Mexico as a control city) with vastly different burdens of urban air pollution. They found that children exposed to the severe air pollution had immune dysregulation with reduced levels of IFN-*γ* and natural killer cells with evidence of elevated systemic inflammation (elevated C-reactive protein and prostaglandin E metabolites).

Indoor air pollution, beyond that of environmental tobacco smoke, which is discussed in a later section, has also been associated with human DIT. Herberth et al. [79] studied the effects of home renovation (e.g., painting, flooring, and new furniture) on inflammatory biomarker profiles of six-year-old children. Significant increases in serum IL-8 and macrophage chemotactic protein 1 (MCP-1) were associated with home renovation activities. Installation of new wall-to-wall carpet gave the strongest single activity association with these markers.

### 5.2. Aluminum

Aluminum exposure during prenatal and childhood development can occur via a variety of routes including via food, certain drugs (aluminum-containing antacids), drinking water, and air [80] including some parenteral nutrition products [81]. The immune system appears to be a sensitive target for aluminum [82]. However, a prevalent opportunity for repeated exposure is alum (aluminum oxyhydroxide)-containing vaccines. Alum is an adjuvant designed to promote a protective immune response, which may include a component of local inflammation (via specific cytokine release). One of the concerns with aluminum is the potential to sometimes induce inappropriate inflammation involving innate immune cells such as macrophages. In some individuals, such as those carrying HLA-DRB1∗01, there appears to be an elevated risk of persistent macrophagic myofasciitis [83, 84], and this link has been proposed as one route to autoimmune/inflammatory syndrome induced by adjuvants (ASIA) [85].

There is evidence to suggest that febrile responses in children following alum-containing vaccination may represent an inflammation-driven hyperresponse that occurs in a subset of children, possibly those possessing certain cytokine gene alleles [86]. A proposed mechanistic basis for alum-induction of DIT in a subpopulation of children was discussed by Terhune and Deth [87]. These authors suggested that the Th2 biasing and inflammasome activating effects of aluminum may present a problem for children carrying genetic variants of certain cytokine genes (e.g., IL-4, IL-13, IL-33, and IL-18). In some subpopulations of children, aluminum adjuvants might enhance the production of nontarget directed IgE thereby elevating the risk of allergy and atopy [87]. Other investigators have suggested that alum may play a role in the induction of Crohn's disease in genetically susceptible individuals [88].

### 5.3. Antibiotics

Antibiotic use in early life has been associated with an elevated risk of immune-based diseases such as childhood asthma. Raciborski et al. [89] found that antibiotic use during the first three years of life was associated with a significantly elevated risk of asthma by 6–8 years of age among 1461 children in Warszawa, Poland. The highest association was found between infants who completed three courses of antibiotic within the first year of life and later childhood asthma (OR = 5.59, 95% CI: 2.6–12.01) [89]. Not all authors agree on this association. Heintze and Petersen [90] argue that various forms of bias weaken the literature on perinatal antibiotic use and risk of childhood asthma. However, the impact of repeated antibiotic use on the microbiome during immune development provides a potential mechanistic basis for DIT, Th2 skewing, and misregulated inflammation [91].

**Figure 1:**
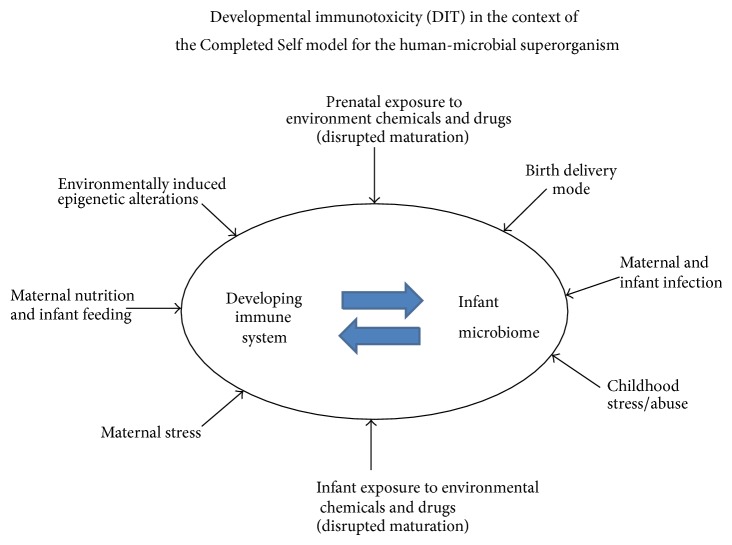
This figure depicts a model following the “Completed Self” paradigm [91] in which the immune system and infant microbiome need to comature without interference or disruption to reduce later-life health risks. The categories of environmental risk factors reported to cause prenatal and/or postnatal disruption are illustrated.

Extensive antibiotic use is of particular concern when viewed in the context of the hygiene hypothesis or the recently-described “Completed Self” model (i.e., where unimpeded comaturation of the development immune system and infant microbiome is critical) [91] (see Figure 1). Under the “Completed Self” paradigm, successful development of a balanced, well-regulated immune system needs co-maturation with a complete microbiome in the infant. The developing immune system receives important signals from the commensal microbes and eventually matures to perceive self as a combination of the mammalian cells and commensal microbes. The successful merger of the infant's mammalian and microbial components into the fully formed human-microbial superorganism may well represent the single most important step distinguishing later-life health from disease. As a result, any prenatal or postnatal environmental exposure that interferes with timely and effective self-completion is a significant health risk [91]. This new immunological view of what constitutes a fully completed infant could impact risk-benefit considerations for antibiotic administration in early life.

### 5.4. Arsenic

Arsenic is found in both inorganic and organic forms. Most of the environmental health concerns have focused on the inorganic forms of arsenic (e.g., arsenite or arsenate) with exposure occurring primarily via ingestion of contaminated food and water and secondarily via inhalation. Some forms of arsenic (e.g., arsenic trioxide) have been used in the treatment of leukemias. The topic of arsenic-induced immunotoxicity was recently reviewed by Dangleben et al. [92]. These authors stressed the increased vulnerability of infants and children to arsenic-induced immune dysfunction and the potential for early-life exposures to produce later-life health problems.

Studies of exposed human populations also suggest that arsenic is a major concern for DIT, and several studies have examined children in Mexico and Bangladesh among highly exposed populations. Soto-Peña et al., [93] found that children in Mexico (6–10 years old) with arsenic exposure primarily from drinking water (evaluated based on urinary levels) had peripheral blood mononuclear cells (PBMs) that were shifted in ex vivo stimulated function (proliferation and cytokine secretion). Rocha-Amador et al. [94] found that children in Mexico living in an area with high exposure to arsenic via drinking water had increased apoptosis among PBMs compared with those from an area with lower exposure levels. In a study of Bangladeshi children where a significant exposure to arsenic can occur via drinking water, Ahmed et al. [95] found that prenatal exposure to arsenic interfered with thymic function affecting T cell development. The proposed route of insult was via oxidative damage and possible misregulation of apoptosis. The same investigators demonstrated that prenatal exposure to arsenic was associated with increased placental inflammation increasing oxidative stress and altering both T cell and cytokine profiles in cord blood [96].

This suggests that, at physiologically-relevant exposures, arsenic-induced DIT can manifest almost immediately during fetal development. This is consistent with the findings of arsenic-exposed children in Mexico where increased arsenic levels were associated with increased basal nitric oxide production by monocytes and increased superoxide anion produced by activated monocytes [97]. Taken together these studies suggest that a proinflammatory state is part of the profile of arsenic-induced human DIT. Lower resistance to certain infectious diseases has been associated with early-life exposure to arsenic. In a prospective population-based cohort study, 1,552 Bangladeshi infants were examined for both lower respiratory tract and diarrhea-associated infections and compared versus maternal arsenic levels during the pregnancy (measured at two time points via urine) [98]. Rahman et al. [98] found that the highest quadril of maternal arsenic exposure versus the lowest had a significantly elevated risk of both forms of mucosal tissue infections. Lower respiratory tract infections had a 69% increased relative risk for infants of high arsenic exposed mothers adjusted relative risk (RR = 1.69; 95% confidence interval (CI), 1.36–2.09), whereas there was a 20% increased risk of diarrheal-associated infections (RR = 1.20; 95% CI, 1.01–1.43) among the same groups.

### 5.5. Bisphenol A

Bisphenol A (BPA) is used in a variety of food and beverage containers. Most human chemical exposure occurs via food and beverages although exposure via air, dust, and water is also possible. Sources of BPA include food storage containers, water bottles, baby bottles, and polycarbonate tableware. BPA has received significant immune system evaluation in recent years although the majority of studies, to date, have been performed in rodents.

Rogers et al. [99] recently reviewed the immunotoxicologic profile of BPA suggesting that it (1) increases Th2 polarization of dendritic cells, (2) alters macrophage inflammatory cytokine production and metabolism but with different dose-dependent effects, (3) decreases T regulatory cells, (4) alters the relative proportions of immunoglobulin (Ig) producing cells, and (5) polarizes CD4+ T helper (Th) cells although the direction of polarization (e.g., Th1 versus Th2) has differed among studies.

Human studies for BPA and DIT are comparatively limited to date. In a National Health and Nutrition Examination Survey (NHANES) study, children and teens less than 18 years of age exhibited an inverse correlation of BPA exposure (urinary levels of BPA) with antibody levels against cytomegalovirus [100]. Kim et al. [101] examined the genomic alteration patterns of Egyptian prepubescent girls (ages 10–13) relative to both genome-wide methylation and methylation of genes previously identified as sensitive to BPA exposure. Among those genes prominently modified were those involved with immune response and autoimmune thyroid disease. Taken together, the animal and human studies suggest that early-life exposure to problematic doses of BPA produces altered gene expression related to immune function and inflammatory responses. However, more research is needed to define the boundaries of these alterations and the impact on various immune-related diseases in later life.

### 5.6. Cesarean Section

Cesarean section (CS) can be a medical necessity in some circumstances. However, the increased prevalence of elective CS (versus vaginal delivery (VD)) has created a public health concern [102]. CS has been reported to alter the course of immune development by producing Th skewing, innate immune dysfunction, and an increased likelihood of exacerbated inflammatory responses (reviewed in [103–105]). There are a minimum of four possible factors with Cesarean delivery that may contribute to subsequent DIT: (1) failure to properly seed the newborn's mucosal tissues with microbiota from the maternal vaginal tract, (2) the prophylactic use of antibiotics, (3) other drug administration connected with the Cesarean operation, and (4) the contrasting placental immune-stress-hormonal environment between the two delivery modes.

In the first category, birth delivery mode can significantly affect the microbiota and the subsequent immune-microbiome interactions. In a Canadian study, Azad et al. [106] found that infants delivered by elective Caesarean were much lower in the bacterial diversity and richness of their microbiome. In the fourth category from above, the immune physiology of vaginal delivery (versus CS) appears to create a strikingly different environment for the full-term fetus. A cross-sectional study of 375 women in The Netherlands compared spontaneous, term VDs versus elective CSs for signs of intrauterine inflammation. Houben et al. [107] found that measures of placental inflammation and amniotic fluid proinflammatory cytokines (IL-6, TNF-*α*, and IL-8) were significantly elevated with VD versus CS. The investigators suggested that increased sterile inflammation during labor and VD delivery may play a critical role in normal parturition and facilitate subsequent maturational processes (e.g., immune and airway maturation) in the newborn [107].

CS has been associated with altered levels of immune cell populations, cytokines, and chemokines in neonates leading Cho and Norman [105] to suggest that it should not be recommended except where there is a clear medical indication or a benefit over risk estimate including long-term consideration for the infant child. For example, CS has been found to skew the infant immune profiles toward a Th2 biased capacity [108]. Innate immune maturational markers are also affected. Elective CS without labor was found to be associated with reduced surface expression of two different toll-like receptors (TLRs): TLR2 and TLR4. In contrast, labor and vaginal delivery appears to upregulate these TLRs to adult levels [109]. Because these TLRs are important in innate immunity, the authors suggest that labor is an important component of ongoing immune maturation [109]. The concentrations of the chemokine, RANTES (CCL5), a chemokine important in recruiting immune cells to inflammatory sites, were found to be lower in neonates from CS than VD [110]. In a prospective study of full-term deliveries, Malamitsi-Puchner et al. [111] found that VD neonates had elevated levels of both soluble IL-2 receptor and TNF-*α* compared with CS delivered babies. Taken together, these studies suggest that neonatal immune profiles, including early inflammatory interactions, are locked into a less mature, more-fetal-like state following CS versus VD deliveries. Not surprisingly, this appears to have consequences relative to risk of childhood chronic diseases.

CS with the outcome of low bacterial diversity in the infant is reported to increase the risk of several immune-based diseases emerging in children including asthma [112, 113], atopic dermatitis [114], celiac disease [115], and type 1 diabetes [116, 117]. A meta-analysis of 23 studies on CS and asthma estimated that the increased risk associated with this birth delivery mode was estimated at 20% [118]. Of note is the observation that specific subpopulations may be at an increased risk for the disease-promoting aspects of CS. For example, Magnus et al. [119] found that the association between CS and childhood asthma (at 3 years of age) was strongest among children of nonatopic mothers.

### 5.7. Childhood Abuse

In children who experience abuse, the developing immune system appears to become wired for dysfunctional responses. In the Nurses' Health Study II, Bertone-Johnson [120] found that women reporting moderate to severe childhood or adolescent abuse had significantly elevated levels of two inflammatory markers CRP and IL-6 as adults. The authors argued that early-life stress may program the immune system for dysregulation and that subsequent immune dysregulation elevates the risk of certain chronic diseases. Slopen et al. [121] make a similar link between childhood adverse experiences, misregulated inflammation, and risk of cardiovascular disease.

### 5.8. Diethylstilbestrol

While human immunological studies on diethylstilbestrol (DES) are limited compared with other health-related studies, there are reports suggesting that prenatally-exposed offspring are at a higher risk of immune-based disease. Overall DES daughters exposed in utero self-reported an increased risk of all immune-based diseases (infections, allergies, and autoimmune conditions). Within specific categories, the women experienced more infectious diseases than non-DES exposed daughters [122]. In a separate study, Strohsnitter et al. [123] examined the incidence of selected autoimmune conditions among DES daughters. They found no overall increase in disease associations for rheumatoid arthritis (RA), systemic lupus erythematosus (SLE), optic neuritis (ON), or idiopathic thrombocytopenic purpura (ITP). However, there was a significant increase in the onset of RA by 45 years of age in the DES-exposed versus nonexposed groups [123].

### 5.9. Ethanol

There are substantial data from animals suggesting that developmental exposure to alcohol produces DIT [55, 124] and can elevate the risk of non-communicable diseases possibly via inflammatory processes [125]. Maternal consumption of alcohol during pregnancy can produce immune-related adverse outcomes in the offspring. In fact, later gestation appears to be particularly sensitive to the effect of ethanol [126]. Among the reported long-term effects was interference with the immune response to influenza virus challenge in mice [127].

Remarkably, human studies are limited for low-level ethanol intake and DIT-related outcomes. Most studies following children exposed in utero to alcohol have focused on growth and behavioral outcomes [128, 129]. Carson et al. [130] utilized the COPSAC prospective birth cohort comprising 411 children born to asthmatic mothers. The children were considered full term and lacked congenital abnormality, systemic illness, or history of mechanical ventilation or lower airway infection. For this study group, the risk of offspring atopic dermatitis was reported to be significantly elevated for any maternal alcohol consumption during pregnancy (HR 1.44, 95% CI 1.05–1.99, *P* = 0.024) even after exclusion of effects of maternal smoking or atopic dermatitis [130].

Two studies reported negative results for maternal alcohol intake and childhood asthma. Yuan et al. [131] examined the incidence of hospitalization for asthma to age 12 among children from 10,440 singleton full-term births in Denmark between the years 1984 and 1987. The authors reported no significant associations between alcohol and no alcohol consumption (HR 0.95; 95% CI 0.70–1.29) including different doses of alcohol as well as binge drinking. In a second study, Shaheen et al. [132] examined maternal alcohol consumption during pregnancy relative to risk of childhood atopic disease (asthma and hayfever) measured at seven years of age within the Avon Longitudinal Study of Parents and Children (ALSPAC). They found no elevated risk for late gestational alcohol consumption with asthma or hayfever and no difference among mothers carrying different alleles for the alcohol dehydrogenase gene [132].

A case-controlled study in Ireland with infants born in 1994–2001 examined factors that are potentially involved with sudden infant death syndrome (SIDS) [133]. McDonnell Naughton et al. [133] reported that mothers of infants with SIDS were more likely to have consumed alcohol during pregnancy than controls (HR 3.59, 95% CI 1.40–9.20).

### 5.10. Lead (Pb)

A cadre of heavy metals has been examined for DIT and associated health risks in both children and adults. Among the most consistent observations with lead (Pb) are elevated risk of oxidative damage and a skewing toward Th2-driven responses with elevated levels of IgE. As an indicator of Pb's ability to produce misregulated inflammation, Pineda-Zavaleta et al. [134] found the macrophages isolated from Pb-exposed children stimulated in vitro with lipopolysaccharide overproduced superoxide anion. Karmaus et al. [135] reported that Pb exposure was associated with elevated IgE in children. Li et al. [136] reported a negative correlation between circulating CD4+T cells and blood lead levels. Lutz et al. [137] found that combined exposure to Pb and environmental tobacco smoke was strongly associated with elevated serum IgE levels in children. The human data are consistent with the animal studies suggesting that Th skewing, increased oxidative stress and tissue damage, and misregulated inflammation are among the adverse immune outcomes following developmental exposure to Pb [138].

### 5.11. Maternal Smoking and Environmental Tobacco Smoke

There are several suggested developmental risk factors for asthma. Among these, maternal smoking during pregnancy and exposure of the infant to environmental tobacco smoke (ETS) were identified by Selgrade et al. [139] as having the most convincing body of evidence connecting environmental exposure to DIT and risk of childhood asthma. Additionally, Prescott [140] identified early life exposure to tobacco smoke producing altered immune function as being an important contributor to risk of allergic diseases. Among the pathways proposed to be involved is the capacity of maternal smoking to alter TLR-mediated responses in infant innate immune cells [140]. Noakes et al. [141] suggest that smoking induced TLR alterations will affect not only the developing immune system but also the “hygiene hypothesis” effects of immune-microbiome interactions in the newborn. The capacity of DIT to disrupt integrity of the immune-microbiome (the Completed Self model) is depicted in Figure 1.

Wilson et al. [142] reported that exposure of children to secondhand smoke produced significant changes in cytokine levels particularly reducing the level of IFN-*γ*. As previously mentioned in the section on Pb, Lutz et al. [137] reported an interaction of environmental risk factors in which Pb-exposed children also exposed to ETS had elevated IgE and IL-4 levels and altered T cell populations. Similar results were obtained by Tebow et al. [143] for exposure covering both prenatal and postnatal periods. These researchers found that parental smoking was associated with a disrupted balance of IFN-*γ* to IL-4 among children of smokers. While IL-4 levels were unchanged in the comparison of children with parental smokers versus non-smokers, reduced IFN-*γ* was associated with parental smoking and a dose response relationship appeared to exist. Therefore, the balance of IFN-*γ* to IL-4 was shifted toward the latter.

Elevated risk of allergic diseases is not the only immune-based concern with early-life exposure to tobacco smoke. Kum-Nji et al. [144] reviewed the literature regarding ETS and childhood infection and concluded that there is no longer a doubt about this association. Supporting evidence has been seen using childhood vaccination. In an examination of 200 infants with a history of parental allergy, Baynam et al. [145] found that, among children with parents who smoked, infants carrying a variant of the IL-4 receptor gene (the IL-4Ralpha 551 QR/QQ genotype) exhibited significantly altered immune responses. These included reduced IgG responses, reductions in certain T cell responses (e.g., those associated with IFN-*γ* production), and altered innate immune (defective TLR-driven) responses upon vaccination with tetanus toxoid. These studies suggest that early-life exposure to smoking causes immune dysbiosis (targeted inappropriately exaggerated responses as well as suppression) that includes both an elevated risk of certain allergic diseases as well as potentially impaired responses to childhood vaccination. In keeping with many other DIT studies involving other risk factors, it also suggests that some human subpopulations are likely to have enhanced vulnerability for smoking-related DIT.

Disrupted immune maturation is not the only pathway through which maternal smoking and ETS appear to affect later-life immune function. Wilhelm-Benartzi [146] found that childhood ETS exposure produced epigenetic marks in genes associated with both immune function and immune signaling.

### 5.12. Paracetamol

Prenatal and early infant exposure to paracetamol (acetaminophen) has been associated with an increased risk of a variety of wheeze-associated disorders in the child including asthma. In the case of prenatal exposure, a study from Denmark examined 197,060 singletons born in northern Denmark in 1996–2008 [147]. Paracetamol exposure during any trimester of the pregnancy resulted in an adjusted odds ratio of 1.35 (95% confidence interval: 1.17–1.57) for risk of asthma by the end of 2009 [131]. For infant exposure, Gonzalez-Barcala et al. [148] studied 20, 000 children in Galicia, Spain, and reported that paracetamol use during the first year of life led to a significant increased risk of asthma in 6-7-year-old children (odds ratio (OR) 2.04 (1.79–2.31)). Henderson and Shaheen [149] recently reviewed the epidemiological data regarding prenatal and infant exposure to paracetamol and an increased risk of childhood asthma. They argue that the evidence is sufficiently strong as to be compelling for this association but also point out that mechanistic causation remains a significant data gap.

One of the potential confounding factors is prevalence of infections and the use of antibiotics, which may coincide with administration of paracetamol [150]. Heintze and Petersen [90] argued that failure to distinguish among the confounding effects of these two factors would significantly weaken the proposed associations. However, Muc et al. [151] performed a cross-sectional study of 1063 primary school children in Portugal in which they partitioned the factors of paracetamol in early childhood and antibiotic administration relative to risk of asthma. Paracetamol use and antibiotic administration were independently found to increase the risk in children of current asthma (at the time of evaluation) as well as ever having asthma. Because frequency of paracetamol use was connected to increased allergic symptoms, the researchers suggested that dose-dependent associations may be present among the data [151]. Not all studies have reported positive associations for paracetamol and asthma. However, based on an understanding of the pathways through which paracetamol is likely to affect offspring immune status and childhood health, Thiele et al. [152] called for a reconsideration of safety and dosage recommendation during pregnancy.

For potential infant use, McBride [153] argued that risk data combined with the likelihood of glutathione depletion by paracetamol in the airways suggested that children at risk for asthma should avoid the use of paracetamol. Selgrade et al. [139] pointed out that accompanying animal data have been generally lacking in DIT models of the human paracetamol-asthma linkage. However, these authors also point to the overall importance of oxidative stress and inflammation as likely routes for xenobiotic-induced, DIT-related asthma. This would be consistent with findings of several research groups.

Evidence from several studies suggests that disruption of effective oxygen species regulation is a likely route to the elevated risk. Kang et al. [154] reported that postnatal pediatric use of paracetamol was more likely to produce asthma among children carrying specific genetic alleles associated with control of oxidative inflammation (*NAT2, Nrf2, *and* GSTP1*). Shaheen et al. [155] examined the effect of specific maternal alleles for nuclear erythroid 2 p45-related factor 2 (*Nrf2*) and glutathione S-transferase (GST) polymorphisms within data from the Avon Longitudinal Study of Parents and Children. They found that maternal *Nrf2* allelic differences had an effect on early gestation exposure to paracetamol and childhood asthma, while the presence of the *GSTT1* allele was important in late gestational exposure to paracetamol [155]. Taken together, these studies suggest that subpopulation differences are likely to exist for the relative risks of association between prenatal exposure to paracetamol and childhood-onset asthma.

### 5.13. Pesticides

Pesticides fall into several different chemical categories (e.g., organophosphate, organochlorine, and pyrethroids). However, humans are likely to be exposed to pesticide mixtures rather than to a single pesticide, and mixtures may result in unanticipated interactions among the pesticides at the molecular level [156]. Human exposure to certain pesticides at sufficient doses has been known to produce a variety of effects on physiological systems with some outcomes potentially linked to their endocrine disrupting activity [157] and altered oxidative stress [158]. In particular, most of the human findings primarily concern early life exposure and childhood neurodevelopmental impairment. In a prospective longitudinal study conducted in the French West Indies, Boucher et al. [159] reported that exposure to the organochlorine pesticide, chlordecone, was associated with impaired neurodevelopment in 18-month-old infants. The effects were seen in boys but not girls.

Three epidemiological studies are significant in pointing to similar conclusions regarding prenatal pesticide exposure and later childhood neurodeficits. In the Columbia University study, Rauh et al. [160] found an inverse association between Working Memory Index and Full-Scale IQ in inner-city children at age seven and the level of prenatal exposure to chlorpyrifos, an organophosphate pesticide. In a Mount Sinai Children's Environmental Health Study, Engel et al. [161] reported that prenatal exposure to organophosphate pesticides was negatively associated with cognitive function by 12 months of age but also continuing later into childhood. In a multi-institutional California study among predominately Latino farmworker families, Bouchard et al. [162] reported that prenatal exposure to organophosphate pesticides was associated with reduced intellectual development at age seven.

Among pesticides, the exposure risks not only involve childhood-onset conditions but also later-life-appearing diseases (e.g., neurodegenerative). Zhou et al. [163] found that early-life exposure of mice to paraquat led to a later silencing in the gene (PINK1) responsible for producing a neuroprotective peptide. At the same time these pesticides activated the brain's innate immune cell resident microglia populations to generate excessive oxidative damage among neurons [164]. The reduced neuroprotection coupled with the increased risk of immune-mediated oxidative damage shifts the equilibrium of the aging brain toward neurodegeneration.

There is a suggestion that pesticide exposure may affect the risk of immune-driven NCDs. In the U.S. Agricultural Health Study, Hoppin et al. [165] found that exposure to pesticides elevated the risk for atopic (but not nonatopic) asthma among farm women. In fact the exposure to pesticides nullified the beneficial effect of growing up on a farm relative to risk of asthma. In this study, a total of 7 of 16 insecticides, 2 of 11 herbicides, and 1 of 4 fungicides were associated with an elevated risk of atopic asthma while permethrin use was the only pesticide associated with an increased risk of nonatopic asthma [165]. The study design [165] did not permit a comparison of differential developmental sensitivities and the potential role of pesticide-induced DIT in risk of asthma. However, the apparent nullification of immune-microbiome protection against asthma (i.e., hygiene hypothesis) raises intriguing questions.

Corsini et al. [166] recently reviewed the literature on pesticides and immunotoxicity. Based on human studies, these investigators concluded that the potential role of pesticides in immunotoxicity is unclear at present. They pointed out the serious limitations of most of the available studies including problems in accessing exposure levels and quite divergent approaches to assessment. The researchers called for better studies that would include pre- and postexposure information and be designed with appropriately matched controls. Beyond the weaknesses discussed by Corsini et al. [166], other weaknesses include a general lack of data regarding early developmental exposures and information regarding potential hypervulnerability for pesticide-induced DIT among human subpopulations.

### 5.14. Polychlorinated Biphenyls

Polychlorinated biphenyls (PCBs) are in a category of persistent organic pollutants (POPs) that can present human health challenges long after release into the environment. Stølevik et al. [167] examined the effects of exposure to PCBs and dioxin among Norwegian mother-child pairs and potential immune effects. They found that exposure to PCBs and dioxins was associated with increased incidence of respiratory infections and reduced antibody response against one (measles) of several childhood vaccinations. This is consistent with the findings of Heilmann et al. [168, 169] who studied perinatal PCB exposure and immune outcomes among children of the Faroe Islands. These researchers reported reduced antibody titers up to age seven to common childhood vaccinations following largely maternal diet-based perinatal exposure to PCBs. For the strongest associations, these investigators found that a doubling of serum PCB concentrations resulted in an approximately 20% reduction in antibody levels. Approximately 28% of the Faroe Islands children were found to be effectively unprotected against the childhood preventable diseases based on the extent of antibody suppression [168, 169].

Significantly for risk of NCDs, Grandjean et al. [170] found that prenatal and lactational exposure of Faroe Island children to marine pollutants including PCBs increased the risk of allergic sensitization. These findings are consistent with the apparent capacity of PCBs to produce disruption of immune homeostasis with effects including not only immunosuppression but also inappropriately enhanced and misdirected immune responses.

### 5.15. Polyfluorinated and Perfluorinated Compounds

The impact of developmental exposure to perfluorooctane sulfonic acid (PFOS) and perfluorooctanoic acid (PFOA) was examined in a prospective cohort birth study in the Faroe Islands [171]. The researchers found that a twofold increase in the levels of PFOS and PFOA at age five resulted in a several-fold increased likelihood of being unprotected against preventable childhood illnesses at age seven. In this case, lack of protection was defined as being below a protective level of antibodies against diphtheria and tetanus [171]. These findings have potentially stark implications for the health protection of children. In fact, the investigators determined that if benchmark dose (BMD) was calculated for the various polyfluorinated compounds using antibody levels as the driver and these were converted to safety limits for PFCs, the current limits may be several hundredfold too high [172].

## 6. Conclusions

DIT and fetal programming are emerging as significant contributors not only to later-life immune dysfunction and misregulated inflammation but also to increased risk of NCDs and particularly chronic diseases. Given the present epidemic of NCDs, the interrelated comorbidities that exist among a myriad of chronic diseases, and the role of NCDs as the greatest cause of death worldwide, better preventative strategies are needed.

Animal model research of DIT dates back several decades and helped to establish the fundamental characteristics surrounding early-life immune vulnerability for later-life disease. Recently, human DIT-related data have shown the relevance of the animal model information concerning dose sensitivity, subpopulation vulnerability, and health ramifications. While data gaps still exist for some categories of environmental risk factors (e.g., bisphenol A, certain pesticides), the way forward seems clear.

Better identification of DIT risk and improved protection of age-, sex-, and genotype-based hypervulnerable subpopulations are needed. This may well require a different approach to safety testing. With the potential for epigenetic alterations to be produced in utero and inheritance of altered immune- and inflammation-related gene expression across generations, it is apparent that efforts to reduce the prevalence of NCDs need to focus on early life. Reducing the prevalence of DIT is an important first step in comprehensive efforts to reduce the prevalence and global impact of NCDs.

## Figures and Tables

**Table 1:** DIT and increased risk of human disease∗.

Disease, disorder, or susceptibility state	Suggested early-life immune-modulating risk factor	Reference(s)
Acute myeloid leukemia	Benzene	[173]
Allergic sensitization	Polychlorinated biphenyls	[170]
Asthma	Maternal paracetamol use	[155]
Atherosclerosis	Maternal hypercholesterolemia	[174]
Atopic dermatitis	Maternal smoking	[175]
Allergic rhinitis	Antibiotics in infancy	[176]
Autism spectrum disorders	Maternal immune activation	[177]
Bipolar disorder	Gestational influenza	[178]
Cardiovascular disease	Childhood abuse	[179]
Celiac disease	Elective cesarean delivery	[180]
Crohn's disease	Maternal smoking	[181]
Chronic obstructive pulmonary disease	Smoke from biomass fuels	[182]
Depression	Childhood trauma	[183]
Endometriosis	Environmental tobacco smoke	[184]
Hypertension	Pesticides (DDT)	[185]
Insulin resistance	Maternal diet	[186]
Lack of protection against diphtheria and tetanus following childhood vaccination	Perfluorinated pollutants	[171]
Multiple sclerosis	Vitamin D insufficiency	[187]
Myalgic encephalomyelitis (Chronic fatigue syndrome)	Childhood trauma	[188, 189]
Narcolepsy (specific subpopulation)	H1N1 flu vaccination	[190, 191]
Obesity/overweight risk	Cesarean delivery	[192]
Otitis media	Maternal smoking/ETS	[193–195]
Parkinson's disease	Pesticides	[164, 196]
Preeclampsia	Traffic-related air pollution	[197]
Psoriasis	Environmental tobacco smoke	[198]
Respiratory infections	Polychlorinated biphenyls	[199, 200]
Rheumatoid arthritis	Maternal smoking	[201]
Schizophrenia	Prenatal immune activation	[202, 203]
Sudden infant death syndrome	Maternal smoking and alcohol consumption	[133]
Type 1 diabetes	Lack of or short-duration breastfeeding	[204]
Ulcerative colitis	Urban living	[205]

^*^This table includes both noncommunicable and communicable diseases and conditions. Environmental risk factors are provided to illustrate an example and are not intended to be an exhaustive listing. The focus is on human studies and data.

## Abbreviations

ALSPAC: Avon Longitudinal Study of Parents and Children
Alum: Aluminum oxyhydroxide
BMD: Benchmark dose
CI: Confidence interval
COPSAC: Copenhagen Prospective Study on Asthma in Childhood
CRP: C-Reactive protein
CS: Cesarean section
CVD: Cardiovascular disease
DES: Diethylstilbestrol
DIT: Developmental immunotoxicity
DOHaD: Developmental origins of health and disease
DZP: Diazepam
HLA: Human leukocyte antigen
Ig: Immunoglobulin
IgE: Immunoglobulin E
IFN-*γ*: Interferon-gamma
Il-4: Interleukin-4
IL-6: Interleukin-6
IL-8: Interleukin-8
NHANES: National Health and Nutrition Examination Survey
NCDs: Noncommunicable diseases
Pb: Lead
PFOA: Perfluorooctanoic acid
PFOS: Perfluorooctane sulfonic acid
POPs: Persistent organic pollutants
RR: Relative risk
Th: T helper
Th2: T helper 2
TBT: Tributyltin oxide
TCDD: 2,3,7,8-Tetrachlorodibenzo-p-dioxin
TLR: Toll-like receptor
TNF-*α*: Tumor necrosis factor-alpha
VD: Vaginal delivery.

## References

[1] Dietert R. R. (2009). Developmental immunotoxicology: focus on health risks. *Chemical Research in Toxicology*.

[2] Dietert R. R., DeWitt J. C., Germolec D. R., Zelikoff J. T. (2010). Breaking patterns of environmentally influenced disease for health risk reduction: Immune perspectives. *Environmental Health Perspectives*.

[3] House R. V., Selgrade M. J. (2010). A quarter-century of immunotoxicology: looking back, looking forward. *Toxicological Sciences*.

[4] Dean J. H., Luster M. I., Boorman G. A. (1982). Methods and approaches for assessing immunotoxicity: an overview. *Environmental Health Perspectives*.

[5] Selgrade M. K., Daniels M. J., Burleson G. R., Lauer L. D., Dean J. H. (1988). Effects of 7,12-dimethylbenz[a]anthracene, benzo[a]pyrene and cyclosporin A on murine cytomegalovirus infection: studies of resistance mechanisms. *International Journal of Immunopharmacology*.

[6] Vos J. G. (2007). Immune suppression as related to toxicology. *Journal of Immunotoxicology*.

[7] Barrett D. J. (1984). Characterization of the acquired immune deficiency syndrome at the cellular and molecular level. *Molecular and Cellular Biochemistry*.

[8] Lane H. C., Fauci A. S. (1985). Immunologic abnormalities in the acquired immunodeficiency syndrome. *Annual Review of Immunology*.

[9] Luster M. I., Portier C., Pait D. G., Germolec D. R. (1994). The use of animal tests in risk assessment for immunotoxicology. *Toxicology in Vitro*.

[10] Luster M. I., Portier C., Pait D. G. (1992). Risk assessment in immunotoxicology. I. Sensitivity and predictability of immune tests. *Fundamental and Applied Toxicology*.

[11] Luster M. I., Portier C., Pait D. G. (1993). Risk assessment in immunotoxicology. II. Relationships between immune and host resistance tests. *Fundamental and Applied Toxicology*.

[12] Vos J. G., Van Loveren H. (1998). Experimental studies on immunosuppression: how do they predict for man?. *Toxicology*.

[13] Basketter D. A., Selbie E., Scholes E. W., Lees D., Kimber I., Botham P. A. (1993). Results with OECD recommend positive control sensitizers in the maximization, Buehler and local lymph node assays. *Food and Chemical Toxicology*.

[14] Basketter D. A., Bremmer J. N., Kammuller M. E. (1994). The identification of chemicals with sensitizing or immunosuppressive properties in routine toxicology. *Food and Chemical Toxicology*.

[15] Luster M. I., Faith R. E., McLachlan J. A. (1978). Alterations of the antibody response following in utero exposure to diethylstilbestrol. *Bulletin of Environmental Contamination and Toxicology*.

[16] Luster M. I., Faith R. E., McLachlan J. A., Clark G. C. (1979). Effect of in utero exposure to diethylstilbestrol on the immune response in mice. *Toxicology and Applied Pharmacology*.

[17] Luster M. I., Faith R. E., Kimmel C. A. (1978). Depression of humoral immunity in rats following chronic developmental lead exposure. *Journal of Environmental Pathology and Toxicology*.

[18] Faith R. E., Luster M. I., Kimmel C. A. (1979). Effect of chronic developmental lead exposure on cell-mediated immune functions. *Clinical and Experimental Immunology*.

[19] Spyker-Cranmer J. M., Barnett J. B., Avery D. L., Cranmer M. F. (1982). Immunoteratology of chlordane: cell-mediated and humoral immune responses in adult mice exposed in utero. *Toxicology and Applied Pharmacology*.

[20] Jagadeesan V., Rukmini C., Vijayaraghavan M., Tulpule P. G. (1982). Immune studies with T-2 toxin: effect of feeding and withdrawal in monkeys. *Food and Chemical Toxicology*.

[21] Dietert R. R., Bloom S. E., Qureshi M. A., Nanna U. C. (1983). Hematological toxicology following embryonic exposure to aflatoxin-B1. *Proceedings of the Society for Experimental Biology and Medicine*.

[22] Dietert R. R., Qureshi M. A., Nanna U. C., Bloom S. E. (1985). Embryonic exposure to aflatoxin-B1: mutagenicity and influence on development and immunity. *Environmental Mutagenesis*.

[23] Urso P., Gengozian N. (1980). Depressed humoral immunity and increased tumor incidence in mice following in utero exposure to benzo[a]pyrene. *Journal of Toxicology and Environmental Health*.

[24] Schlumpf M., Bütikofer E. E., Schreiber A. A., Parmar R., Ramseier H. R., Lichtensteiger W. (1994). Delayed developmental immunotoxicity of prenatal benzodiazepines. *Toxicology in Vitro*.

[25] Holladay S. D., Smith B. J. (1995). Alterations in murine fetal thymus and liver hematopoietic cell populations following developmental exposure to 7,12- dimethylbenz[a]anthracene. *Environmental Research*.

[26] Selevan S. G., Kimmel C. A., Mendola P. (2000). Identifying critical windows of exposure for children’s health. *Environmental Health Perspectives*.

[27] Holladay S. D., Smialowicz R. J. (2000). Development of the murine and human immune system: differential effects of immunotoxicants depend on time of exposure. *Environmental Health Perspectives*.

[28] Dietert R. R., Etzel R. A., Chen D. (2000). Workshop to identify critical windows of exposure for children’s health: immune and respiratory systems work group summary. *Environmental Health Perspectives*.

[29] Holladay S. D. (2004). *Developmental Immunotoxicology*.

[30] Langley-Evans S. (1997). Fetal programming of immune function and respiratory disease. *Clinical and Experimental Allergy*.

[31] Ward A. M. V., Phillips D. I. W. (2001). Fetal programming of stress responses. *Stress*.

[32] Barker D. J. P. (2005). The developmental origins of insulin resistance. *Hormone Research*.

[33] Luebke R. W., Chen D. H., Dietert R., Yang Y., King M., Luster M. I. (2006). The comparative immunotoxicity of five selected compounds following developmental or adult exposure. *Journal of Toxicology and Environmental Health B*.

[34] Dewitt J. C., Peden-Adams M. M., Keil D. E., Dietert R. R. (2012). Current status of developmental immunotoxicity: early-life patterns and testing. *Toxicologic Pathology*.

[35] Dietert R. R., Holsapple M. P. (2007). Methodologies for developmental immunotoxicity (DIT) testing. *Methods*.

[36] Collinge M., Burns-Naas L. A., Chellman G. J. (2012). Developmental immunotoxicity (DIT) testing of pharmaceuticals: current practices, state of the science, knowledge gaps, and recommendations. *Journal of Immunotoxicology*.

[37] Piersma A. H., Tonk E. C., Makris S. L., Crofton K. M., Dietert R. R., van Loveren H. (2012). Juvenile toxicity testing protocols for chemicals. *Reproductive Toxicology*.

[38] Dietert R. R., Piepenbrink M. S. (2006). Perinatal immunotoxicity: why adult exposure assessment fails to predict risk. *Environmental Health Perspectives*.

[39] Dietert R. R., DeWitt J. C., Luebke R. W., Dietert R. R., Dietert R. W. (2012). Reducing the prevalence of immune-based chronic dsisease. *Immunotoxicity, Immune Dysfunction, and Chronic Disease*.

[40] Dietert R. R. (2011). Role of developmental immunotoxicity and immune dysfunction in chronic disease and cancer. *Reproductive Toxicology*.

[41] Barker D. J. P., Winter P. D., Osmond C., Margetts B., Simmonds S. J. (1989). Weight in infancy and death from ischaemic heart disease. *The Lancet*.

[42] Barker D. J. P., Bull A. R., Osmond C., Simmonds S. J. (1990). Fetal and placental size and risk of hypertension in adult life. *British Medical Journal*.

[43] Barker D. J. (1991). The intrauterine environment and adult cardiovascular disease. *Ciba Foundation Symposium*.

[44] Paneth N., Susser M. (1995). Early origin of coronary heart disease (the “Barker hypothesis”). Hypotheses, no matter how intriguing, need rigorous attempts at refutation. *British Medical Journal*.

[45] Wendy E H., Rees M., Kile E., Mathews J. D., Wang Z. (1999). A new dimension to the Barker hypothesis: low birthweight and susceptibility to renal disease. *Kidney International*.

[46] Holness M. J., Langdown M. L., Sugden M. C. (2000). Early-life programming of susceptibility to dysregulation of glucose metabolism and the development of type 2 diabetes mellitus. *Biochemical Journal*.

[47] Darney S., Fowler B., Grandjean P., Heindel J., Mattison D., Slikker W. (2011). Prenatal Programming and Toxicity II (PPTOX II): role of environmental stressors in the developmental origins of disease. *Reproductive Toxicology*.

[48] Barouki R., Gluckman P. D., Grandjean P., Hanson M., Heindel J. J. (2012). Developmental origins of non-communicable disease: implications for research and public health. *Environmental Health*.

[49] DeWitt J. C., Peden-Adams M. M., Keil D. E., Dietert R. R. (2012). Developmental immunotoxicity (DIT): assays for evaluating effects of exogenous agents on development of the immune system. *Current Protocols in Toxicology*.

[50] Burleson G. R., Burleson F. G. (2008). Testing human biologicals in animal host resistance models. *Journal of Immunotoxicology*.

[51] Collinge M., Thorn M., Peachee V., White K. (2013). Validation of a Candida albicans delayed-type hypersensitivity (DTH) model in female juvenile rats for use in immunotoxicity assessments. *Journal of Immunotoxicology*.

[52] Tonk E. C. M., de Groot D. M. G., Penninks A. H. (2010). Developmental immunotoxicity of methylmercury: the relative sensitivity of developmental and immune parameters. *Toxicological Sciences*.

[53] Tonk E. C. M., Verhoef A., de la Fonteyne L. J. J. (2011). Developmental immunotoxicity in male rats after juvenile exposure to di-n-octyltin dichloride (DOTC). *Reproductive Toxicology*.

[54] Tonk E. C. M., Verhoef A., Gremmer E. R., van Loveren H., Piersma A. H. (2012). Relative sensitivity of developmental and immune parameters in juvenile versus adult male rats after exposure to di(2-ethylhexyl) phthalate. *Toxicology and Applied Pharmacology*.

[55] Tonk E. C., Verhoef A., Gremmer E. R., van Loveren H., Piersma A. H. (2013). Developmental immunotoxicity in male rats after juvenile exposure to ethanol. *Toxicology*.

[56] Dietert R. R., Lee J.-E., Olsen J., Fitch K., Marsh J. A. (2003). Developmental immunotoxicity of dexamethasone: comparison of fetal versus adult exposures. *Toxicology*.

[57] Gehrs B. C., Riddle M. M., Williams W. C., Smialowicz R. J. (1997). Alterations in the developing immune system of the F344 rat after perinatal exposure to 2, 3, 7, 8-tetrachlorodibenzo-p-dioxin: II. Effects on the pup and the adult. *Toxicology*.

[58] Miller T. E., Golemboski K. A., Ha R. S., Bunn T., Sanders F. S., Dietert R. R. (1998). Developmental exposure to lead causes persistent immunotoxicity in Fischer 344 rats. *Toxicological Sciences*.

[59] Walker D. B., Williams W. C., Copeland C. B., Smialowicz R. J. (2004). Persistent suppression of contact hypersensitivity, and altered T-cell parameters in F344 rats exposed perinatally to 2,3,7,8-tetrachlorodibenzo-p- dioxin (TCDD). *Toxicology*.

[60] Hussain I., Piepenbrink M. S., Fitch K. J., Marsh J. A., Dietert R. R. (2005). Developmental immunotoxicity of cyclosporin-A in rats: age-associated differential effects. *Toxicology*.

[61] Bunn T. L., Parsons P. J., Kao E., Dietert R. R. (2001). Exposure to lead during critical windows of embryonic development: differential immunotoxic outcome based on stage of exposure and gender. *Toxicological Sciences*.

[62] Jenmalm M. C., Duchén K. (2013). Timing of allergy-preventive and immunomodulatory dietary interventions—are prenatal, perinatal or postnatal strategies optimal?. *Clinical and Experimental Allergy*.

[63] Bloom D. E., Cafiero E. T., Jané-Llopis E. (2011). *The Global Economic Burden of Noncommunicable Diseases*.

[64] Dietert R. R. (2012). Misregulated inflammation as an outcome of early-life exposure to endocrine-disrupting chemicals. *Reviews in Environmental Health*.

[65] Prescott S. L. (2013). Early-life environmental determinants of allergic diseases and the wider pandemic of inflammatory noncommunicable diseases. *Journal of Allergy and Clinical Immunology*.

[66] Dietert R. R., Dietert R. W. (2012). *Immunotoxicity, Immune Dysfunction, and Chronic Disease*.

[67] Dietert R. R., Zelikoff J. T. (2009). Pediatric immune dysfunction and health risks following early-life immune insult. *Current Pediatric Reviews*.

[68] Dietert R. R., Zelikoff J. T. (2010). Identifying patterns of immune-related disease: use in disease prevention and management. *World Journal of Pediatrics*.

[69] Dietert R. R., Weiss B. (2012). Immune system disorders. *Aging and Vulnerability to Environmental Chemicals: Age-Related Disorders and Their Origins in Environmental Exposures*.

[70] Pham-Short A., Donaghue K. C., Ambler G., Chan A. K., Craig M. E. (2012). Coeliac disease in Type 1 diabetes from 1990 to 2009: higher incidence in young children after longer diabetes duration. *Diabetic Medicine*.

[71] Greco D., Pisciotta M., Gambina F., Maggio. F. (2011). Graves’ disease in subjects with type 1 diabetes mellitus: a prevalence study in western Sicily (Italy). *Primary Care Diabetes*.

[72] Papoutsakis C., Priftis K. N., Drakouli M. (2013). Childhood overweight/obesity and asthma: is there a link? A systematic review of recent epidemiologic evidence. *Journal of the Academy of Nutrition and Dietetics*.

[73] Lazaratou H., Soldatou A., Dikeos D. (2012). Medical comorbidity of sleep disorders in children and adolescents. *Current Opinion in Psychiatry*.

[74] Tanaka M., Anders S., Kinney D. K., Dietert R. R., Dietert R. W. (2012). Environment, the immune system, and depression: an integrative review and discussion of the infection-defense hypothesis. *Immunotoxicity, Immune Dysfunction, and Chronic Disease*.

[75] Anders S., Tanaka M., Kinney D. K. (2013). Depression as an evolutionary strategy for defense against infection. *Brain, Behavior, and Immunity*.

[76] Nadeau K., McDonald-Hyman C., Noth E. M. (2010). Ambient air pollution impairs regulatory T-cell function in asthma. *Journal of Allergy and Clinical Immunology*.

[77] Kerkhof M., Postma D. S., Brunekreef B. (2010). Toll-like receptor 2 and 4 genes influence susceptibility to adverse effects of traffic-related air pollution on childhood asthma. *Thorax*.

[78] Calderón-Garcidueñas L., Macías-Parra M., Hoffmann H. J. (2009). Immunotoxicity and environment: immunodysregulation and systemic inflammation in children. *Toxicological Pathology*.

[79] Herberth G., Gubelt R., Röder S. (2009). Increase of inflammatory markers after indoor renovation activities: The LISA Birth Cohort Study. *Pediatric Allergy and Immunology*.

[80] Krewski D., Yokel R. A., Nieboer E. (2007). Human health risk assessment for aluminium, aluminium oxide, and aluminium hydroxide. *Journal of Toxicology and Environmental Health B*.

[81] Poole R. L., Pieroni K. P., Gaskari S., Dixon T., Kerner J. A. (2012). Aluminum exposure in neonatal patients using the least contaminated parenteral nutrition solution products. *Nutrients*.

[82] Zhu Y. Z., Liu D. W., Liu Z. Y., Li Y. F. (2013). Impact of aluminum exposure on the immune system: a mini review. *Environmental Toxicology and Pharmacology*.

[83] Israeli E., Agmon-Levin N., Blank M., Shoenfeld Y. (2011). Macrophagic myofaciitis a vaccine (alum) autoimmune-related disease. *Clinical Reviews in Allergy and Immunology*.

[84] Gherardi R. K., Authier F. J. (2012). Macrophagic myofasciitis: characterization and pathophysiology. *Lupus*.

[85] Vera-Lastra O., Medina G., Cruz-Dominguez Mdel P., Jara L. J., Shoenfeld Y. (2013). Autoimmune/inflammatory syndrome induced by adjuvants (Shoenfeld's syndrome): clinical and immunological spectrum. *Expert Reviews in Clinical Immunology*.

[86] Nakayama T., Kashiwagi Y., Kawashima H., Kumagai T., Ishii K. J., Ihara T. (2012). Alum-adjuvanted H5N1 whole virion inactivated vaccine (WIV) enhanced inflammatory cytokine productions. *Vaccine*.

[87] Terhune T. D., Deth R. C. (2013). How aluminum adjuvants could promote and enhance non-target IgE synthesis in a genetically-vulnerable sub-population. *Journal of Immunotoxicology*.

[88] Lerner A. (2012). Aluminum as an adjuvant in Crohn’s disease induction. *Lupus*.

[89] Raciborski F., Tomaszewska A., Komorowski J. (2012). The relationship between antibiotic therapy in early childhood and the symptoms of allergy in children aged 6–8 years—the questionnaire study results. *International Journal of Occupational Medicine and Environmental Health*.

[90] Heintze K., Petersen K. U. (2013). The case of drug causation of childhood asthma: antibiotics and paracetamol. *European Journal of Clinical Pharmacology*.

[91] Dietert R. R., Dietert J. M. (2012). The completed self: an immunological view of the human-microbiome superorganism and risk of chronic diseases. *Entropy*.

[92] Dangleben N. L., Skibola C. F., Smith M. T. (2013). Arsenic immunotoxicity: a review. *Environmental Health*.

[93] Soto-Peña G. A., Luna A. L., Acosta-Saavedra L. (2006). Assessment of lymphocyte subpopulations and cytokine secretion in children exposed to arsenic. *FASEB Journal*.

[94] Rocha-Amador D. O., Calderón J., Carrizales L., Costilla-Salazar R., Pérez-Maldonado I. N. (2011). Apoptosis of peripheral blood mononuclear cells in children exposed to arsenic and fluoride. *Environmental Toxicology and Pharmacology*.

[95] Ahmed S., Ahsan K. B., Kippler M. (2012). In utero arsenic exposure is associated with impaired thymic function in newborns possibly via oxidative stress and apoptosis. *Toxicological Sciences*.

[96] Ahmed S., Khoda S. M.-E., Rekha R. S. (2011). Arsenic-associated oxidative stress, inflammation, and immune disruption in human placenta and cord blood. *Environmental Health Perspectives*.

[97] Luna A. L., Acosta-Saavedra L. C., Lopez-Carrillo L. (2010). Arsenic alters monocyte superoxide anion and nitric oxide production in environmentally exposed children. *Toxicology and Applied Pharmacology*.

[98] Rahman A., Vahter M., Ekström E.-C., Persson L.-Å. (2011). Arsenic exposure in pregnancy increases the risk of lower respiratory tract infection and diarrhea during infancy in Bangladesh. *Environmental Health Perspectives*.

[99] Rogers J. A., Metz L., Yong V. W. (2013). Review: endocrine disrupting chemicals and immune responses: a focus on bisphenol-A and its potential mechanisms. *Molecular Immunology*.

[100] Rees Clayton E. M., Todd M., Dowd J. B., Aiello A. E. (2011). The impact of bisphenol A and triclosan on immune parameters in the U.S. population, NHANES 2003–2006. *Environmental Health Perspectives*.

[101] Kim J. H., Rozek L. S., Soliman A. S. (2013). Bisphenol A-associated epigenomic changes in prepubescent girls: a cross-sectional study in Gharbiah, Egypt. *Environmental Health*.

[102] Unterscheider J., McMenamin M., Cullinane F. (2011). Rising rates of caesarean deliveries at full cervical dilatation: a concerning trend. *European Journal of Obstetrics Gynecology and Reproductive Biology*.

[103] Neu J., Rushing J. (2011). Cesarean Versus Vaginal Delivery: long-term infant outcomes and the Hygiene Hypothesis. *Clinics in Perinatology*.

[104] Dietert R. R. (2013). Natural childbirth and breastfeeding as preventive measures of immune-microbiome dysbiosis and misregulated inflammation. *Journal of Ancient Diseases & Preventive Remedies*.

[105] Cho C. E., Norman M. (2013). Cesarean section and development of the immune system in the offspring. *American Journal of Obstetrics and Gynecology*.

[106] Azad M. B., Konya T., Maughan H. (2013). Gut microbiota of healthy Canadian infants: profiles by mode of delivery and infant diet at 4 months. *Canadian Medical Association Journal*.

[107] Houben M. L., Nikkels P. G. J., van Bleek G. M. (2009). The association between intrauterine inflammation and spontaneous vaginal delivery at term: A Cross-Sectional Study. *PLoS ONE*.

[108] Romero V. C., Somers E. C., Stolberg V. (2013). Developmental programming for allergy: a secondary analysis of the Mothers, Omega-3, and Mental Health Study. *American Journal of Obstetrics & Gynecology*.

[109] Shen C.-M., Lin S.-C., Niu D.-M., Kou Y. R. (2009). Labour increases the surface expression of two toll-like receptors in the cord blood monocytes of healthy term newborns. *Acta Paediatrica, International Journal of Paediatrics*.

[110] Królak-Olejnik B., Olejnik I. (2012). Late-preterm cesarean delivery and chemokines concentration in the umbilical cord blood of neonates. *Journal of Maternal-Fetal and Neonatal Medicine*.

[111] Malamitsi-Puchner A., Protonotariou E., Boutsikou T., Makrakis E., Sarandakou A., Creatsas G. (2005). The influence of the mode of delivery on circulating cytokine concentrations in the perinatal period. *Early Human Development*.

[112] Roduit C., Scholtens S., De Jongste J. C. (2009). Asthma at 8 years of age in children born by caesarean section. *Thorax*.

[113] Azad M. B., Kozyrskyj A. L. (2012). Perinatal programming of asthma: the role of gut microbiota. *Clinical and Developmental Immunology*.

[114] Abrahamsson T. R., Jakobsson H. E., Andersson A. F., Björkstén B., Engstrand L., Jenmalm M. C. (2012). Low diversity of the gut microbiota in infants with atopic eczema. *Journal of Allergy and Clinical Immunology*.

[115] Decker E., Hornef M., Stockinger S. (2011). Cesarean delivery is associated with celiac disease but not inflammatory bowel disease in children. *Gut Microbes*.

[116] Phillips J., Gill N., Sikdar K., Penney S., Newhook L. A. (2012). History of cesarean section associated with childhood onset of T1DM in Newfoundland and Labrador, Canada. *Journal of Environmental and Public Health*.

[117] Bonifacio E., Warncke K., Winkler C., Wallner M., Ziegler A.-G. (2011). Cesarean section and interferon-induced helicase gene polymorphisms combine to increase childhood type 1 diabetes risk. *Diabetes*.

[118] Thavagnanam S., Fleming J., Bromley A., Shields M. D., Cardwell C. R. (2008). A meta-analysis of the association between Caesarean section and childhood asthma. *Clinical and Experimental Allergy*.

[119] Magnus M. C., Håberg S. E., Stigum H. (2011). Delivery by Cesarean section and early childhood respiratory symptoms and disorders: the Norwegian mother and child cohort study. *American Journal of Epidemiology*.

[120] Bertone-Johnson E. R., Whitcomb B. W., Missmer S. A., Karlson E. W., Rich-Edwards J. W. (2012). Inflammation and early-life abuse in women. *American Journal of Preventive Medicine*.

[121] Slopen N., Koenen K. C., Kubzansky L. D. (2012). Childhood adversity and immune and inflammatory biomarkers associated with cardiovascular risk in youth: a systematic review. *Brain, Behavior, and Immunity*.

[122] Vingerhoets A. J. J. M., Assies J., Goodkin K., Van Heck G. L., Bekker M. H. (1998). Prenatal diethylstilbestrol exposure and self-reported immune-related diseases. *European Journal of Obstetrics Gynecology and Reproductive Biology*.

[123] Strohsnitter W. C., Noller K. L., Troisi R. (2010). Autoimmune disease incidence among women prenatally exposed to diethylstilbestrol. *Journal of Rheumatology*.

[124] Tonk E. C., de Groot D. M., Wolterbeek A. P. (2013). Developmental immunotoxicity of ethanol in an extended one-generation reproductive toxicity study. *Archives of Toxicology*.

[125] Zhang X., Lan N., Bach P. (2012). Prenatal alcohol exposure alters the course and severity of adjuvant-induced arthritis in female rats. *Brain, Behavior, and Immunity*.

[126] Ping X.-D., Harris F. L., Brown L. A. S., Gauthier T. W. (2007). In vivo dysfunction of the term alveolar macrophage after in utero ethanol exposure. *Alcoholism*.

[127] McGill J., Meyerholz D. K., Edsen-Moore M. (2009). Fetal exposure to ethanol has long-term effects on the severity of influenza virus infections. *Journal of Immunology*.

[128] Kelly S. J., Day N., Streissguth A. P. (2000). Effects of prenatal alcohol exposure on social behavior in humans and other species. *Neurotoxicology and Teratology*.

[129] Day N. L., Helsel A., Sonon K., Goldschmidt L. (2013). The association between prenatal alcohol exposure and behavior at 22 years of age. *Alcoholism: Clinical and Experimental Research*.

[130] Carson C. G., Halkjaer L. B., Jensen S. M., Bisgaard H. (2012). Alcohol intake in pregnancy increases the child's risk of atopic dermatitis. The COPSAC prospective birth cohort study of a high risk population. *PLoS One*.

[131] Yuan W., Sørensen H. T., Basso O., Olsen J. (2004). Prenatal maternal alcohol consumption and hospitalization with asthma in childhood: A Population-Based Follow-up Study. *Alcoholism*.

[132] Shaheen S. O., Rutterford C., Zuccolo L. (2013). Prenatal alcohol exposure and childhood atopic disease: a Mendelian randomization approach. *Journal of Allergy and Clinical Immunology*.

[133] McDonnell Naughton M., McGarvey C., Regan M. O., Matthews T. (2012). Maternal smoking and alcohol consumption during pregnancy as risk factors for sudden infant death. *Irish Medical Journal*.

[134] Pineda-Zavaleta A. P., García-Vargas G., Borja-Aburto V. H. (2004). Nitric oxide and superoxide anion production in monocytes from children exposed to arsenic and lead in region Lagunera, Mexico. *Toxicology and Applied Pharmacology*.

[135] Karmaus W., Brooks K. R., Nebe T., Witten J., Obi-Osius N., Kruse H. (2005). Immune function biomarkers in children exposed to lead and organochlorine compounds: A Cross-Sectional Study. *Environmental Health*.

[136] Li S., Zhengyan Z., Rong L. I., Hanyun C. (2005). Decrease of CD4+ T-lymphocytes in children exposed to environmental lead. *Biological Trace Element Research*.

[137] Lutz P. M., Kelty E. A., Brown T. D., Wilson T. J., Brock G., Neal R. E. (2012). Environmental cigarette smoke exposure modulates IgE levels of Pb-exposed children. *Toxicology*.

[138] Leifer C. A., Dietert R. R. (2011). Early life environment and developmental immunotoxicity in inflammatory dysfunction and disease. *Toxicological and Environmental Chemistry*.

[139] Selgrade M. K., Blain R. B., Fedak K. M., Cawley M. A. (2013). Potential risk of asthma associated with in utero exposure to xenobiotics. *Birth Defects Research C*.

[140] Prescott S. L. (2008). Effects of early cigarette smoke exposure on early immune development and respiratory disease. *Paediatric Respiratory Reviews*.

[141] Noakes P. S., Hale J., Thomas R., Lane C., Devadason S. G., Prescott S. L. (2006). Maternal smoking is associated with impaired neonatal toll-like-receptor-mediated immune responses. *European Respiratory Journal*.

[142] Wilson K. M., Wesgate S. C., Pier J. (2012). Secondhand smoke exposure and serum cytokine levels in healthy children. *Cytokine*.

[143] Tebow G., Sherrill D. L., Lohman I. C. (2008). Effects of parental smoking on interferon *γ* production in children. *Pediatrics*.

[144] Kum-Nji P., Meloy L., Herrod H. G. (2006). Environmental tobacco smoke exposure: prevalence and mechanisms of causation of infections in children. *Pediatrics*.

[145] Baynam G., Khoo S.-K., Rowe J. (2007). Parental smoking impairs vaccine responses in children with atopic genotypes. *Journal of Allergy and Clinical Immunology*.

[146] Wilhelm-Benartzi C. S., Christensen B. C., Koestler D. C. (2011). Association of secondhand smoke exposures with DNA methylation in bladder carcinomas. *Cancer Causes and Control*.

[147] Andersen A. B. T., Farkas D. K., Mehnert F., Ehrenstein V., Erichsen R. (2012). Use of prescription paracetamol during pregnancy and risk of asthma in children: a population-based Danish cohort study. *Clinical Epidemiology*.

[148] Gonzalez-Barcala F. J., Pertega S., Castro T. P. (2013). Exposure to paracetamol and asthma symptoms. *European Journal of Public Health*.

[149] Henderson A. J., Shaheen S. O. (2013). Acetaminophen and asthma. *Paediatric Respiratory Reviews*.

[150] Scialli A. R., Ang R., Breitmeyer J., Royal M. A. (2010). Childhood asthma and use during pregnancy of acetaminophen: a critical review. *Reproductive Toxicology*.

[151] Muc M., Padez C., Pinto A. M. (2013). Exposure to paracetamol and antibiotics in early life and elevated risk of asthma in childhood. *Advances in Experimental Medicine and Biology*.

[152] Thiele K., Kessler T., Arck P., Erhardt A., Tiegs G. (2013). Acetaminophen and pregnancy: short- and long-term consequences for mother and child. *Journal of Reproductive Immunology*.

[153] McBride J. T. (2011). The association of acetaminophen and asthma prevalence and severity. *Pediatrics*.

[154] Kang S. H., Jung Y. H., Kim H. Y. (2013). Effect of paracetamol use on the modification of the development of asthma by reactive oxygen species genes. *Annuals of Allergy, Asthma & Immunology*.

[155] Shaheen S. O., Newson R. B., Ring S. M., Rose-Zerilli M. J., Holloway J. W., Henderson A. J. (2010). Prenatal and infant acetaminophen exposure, antioxidant gene polymorphisms, and childhood asthma. *Journal of Allergy and Clinical Immunology*.

[156] Hernández A. F., Parrón T., Tsatsakis A. M., Requena M., Alarcón R., López-Guarnido O. (2013). Toxic effects of pesticide mixtures at a molecular level: their relevance to human health. *Toxicology*.

[157] Kjeldsen L. S., Ghisari M., Bonefeld-Jørgensen E. C. (2013). Currently used pesticides and their mixtures affect the function of sex hormone receptors and aromatase enzyme activity. *Toxicology and Applied Pharmacology*.

[158] Bonvallot N., Tremblay-Franco M., Chevrier C. (2013). Metabolomics tools for describing complex pesticide exposure in pregnant women in brittany (france). *PLoS One*.

[159] Boucher S., Simard M. N., Muckle G. (2013). Exposure to an organochlorine pesticide (chlordecone) and development of 18-month-old infants. *Neurotoxicology*.

[160] Rauh V., Arunajadai S., Horton M. (2011). Seven-year neurodevelopmental scores and prenatal exposure to chlorpyrifos, a common agricultural pesticide. *Environmental Health Perspectives*.

[161] Engel S. M., Wetmur J., Chen J. (2011). Prenatal exposure to organophosphates, paraoxonase 1, and cognitive development in childhood. *Environmental Health Perspectives*.

[162] Bouchard M. F., Chevrier J., Harley K. G. (2011). Prenatal exposure to organophosphate pesticides and IQ in 7-year-old children. *Environmental Health Perspectives*.

[163] Zhou H., Huang C., Tong J., Xia X.-G. (2011). Early exposure to paraquat sensitizes dopaminergic neurons to subsequent silencing of PINK1 gene expression in mice. *International Journal of Biological Sciences*.

[164] Taetzsch T., Block M. L. (2013). Pesticides, microglial NOX2, and Parkinson's disease. *Journal of Biochemical and Molecular Toxicology*.

[165] Hoppin J. A., Umbach D. M., London S. J. (2008). Pesticides and atopic and nonatopic asthma among farm women in the agricultural health study. *American Journal of Respiratory and Critical Care Medicine*.

[166] Corsini E., Sokooti M., Galli C. L., Moretto A., Colosiom C. (2013). Pesticide induced immunotoxicity in humans: a comprehensive review of the existing evidence. *Toxicology*.

[167] Stølevik S. B., Nygaard U. C., Namork E. (2013). Prenatal exposure to polychlorinated biphenyls and dioxins from the maternal diet may be associated with immunosuppressive effects that persist into early childhood. *Food and Chemical Toxicology*.

[168] Heilmann C., Grandjean P., Weihe P., Nielsen F., Budtz-Jørgensen E. (2006). Reduced antibody responses to vaccinations in children exposed to polychlorinated biphenyls. *PLoS Medicine*.

[169] Heilmann C., Budtz-Jørgensen E., Nielsen F., Heinzow B., Weihe P., Grandjean P. (2010). Serum concentrations of antibodies against vaccine toxoids in children exposed perinatally to immunotoxicants. *Environmental Health Perspectives*.

[170] Grandjean P., Poulsen L. K., Heilmann C., Steuerwald U., Weihe P. (2010). Allergy and sensitization during childhood associated with prenatal and lactational exposure to marine pollutants. *Environmental Health Perspectives*.

[171] Grandjean P., Andersen E. W., Budtz-Jørgensen E. (2012). Serum vaccine antibody concentrations in children exposed to perfluorinated compounds. *Journal of the American Medical Association*.

[172] Grandjean P., Budtz-Jørgensen E. (2013). Immunotoxicity of perfluorinated alkylates: calculation of benchmark doses based on serum concentrations in children. *Environmental Health*.

[173] Vinceti M., Rothman K. J., Crespi C. M. (2012). Leukemia risk in children exposed to benzene and PM10 from vehicular traffic: a case-control study in an Italian population. *European Journal of Epidemiology*.

[174] Palinski W., Yamashita T., Freigang S., Napoli C. (2007). Developmental programming: maternal hypercholesterolemia and immunity influence susceptibility to atherosclerosis. *Nutrition Reviews*.

[175] Hinz D., Bauer M., Röder S. (2012). Cord blood Tregs with stable FOXP3 expression are influenced by prenatal environment and associated with atopic dermatitis at the age of one year. *Allergy*.

[176] Kim W. K., Kwon J. W., Seo J. H. (2012). Interaction between IL13 genotype and environmental factors in the risk for allergic rhinitis in Korean children. *Journal of Allergy and Clinical Immunology*.

[177] Gesundheit B., Rosenzweig J. P., Naor D. (2013). Immunological and autoimmune considerations of Autism Spectrum Disorders. *Journal Autoimmunity*.

[178] Parboosing R., Ba Y., Shen L., Schaefer C. A., Brown A. S. (2013). Gestational influenza and bipolar disorder in adult offspring. *Journal of the American Medical Association Psychatry*.

[179] Hosang G. M., Johnson S. L., Kiecolt-Glaser J. (2013). Gender specific association of child abuse and adult cardiovascular disease in a sample of patients with basal cell carcinoma. *Child Abuse & Neglect*.

[180] Mårild K., Stephansson O., Montgomery S., Murray J. A., Ludvigsson J. F. (2012). Pregnancy outcome and risk of celiac disease in offspring: a nationwide case-control study. *Gastroenterology*.

[181] Roberts S. E., Wotton C. J., Williams J. G., Griffith M., Goldacre M. J. (2011). Perinatal and early life risk factors for inflammatory bowel disease. *World Journal of Gastroenterology*.

[182] Kodgule R., Salvi S. (2012). Exposure to biomass smoke as a cause for airway disease in women and children. *Current Opinion in Allergy and Clinical Immunology*.

[183] Lu S., Peng H., Wang L. (2013). Elevated specific peripheral cytokines found in major depressive disorder patients with childhood trauma exposure: a cytokine antibody array analysis. *Comprehensive Psychiatry*.

[184] Kvaskoff M., Bijon A., Clavel-Chapelon F., Mesrine S., Boutron-Ruault M. C. (2013). Childhood and adolescent exposures and the risk of endometriosis. *Epidemiology*.

[185] La Merrill M., Cirillo P. M., Terry M. B., Krigbaum N. Y., Flom J. D., Cohn B. A. (2013). Prenatal exposure to the pesticide DDT and hypertension diagnosed in women before age 50: A Longitudinal Birth Cohort Study. *Environmental Health Perspective*.

[186] Thompson J. A., Regnault T. R. H. (2011). In utero origins of adult insulin resistance and vascular dysfunction. *Seminars in Reproductive Medicine*.

[187] Koch M. W., Metz L. M., Agrawal S. M., Yong V. W. (2013). Environmental factors and their regulation of immunity in multiple sclerosis. *Journal of Neurological Sciences*.

[188] Maes M., Twisk F. N. M., Kubera M., Ringel K. (2012). Evidence for inflammation and activation of cell-mediated immunity in Myalgic Encephalomyelitis/Chronic Fatigue Syndrome (ME/CFS): increased interleukin-1, tumor necrosis factor-*α*, PMN-elastase, lysozyme and neopterin. *Journal of Affective Disorders*.

[189] Kempke S., Luyten P., Claes S. (2013). The prevalence and impact of early childhood trauma in Chronic Fatigue Syndrome. *Journal of Psychiatric Research*.

[190] Szakács A., Darin N., Hallböök T. (2013). Increased childhood incidence of narcolepsy in western Sweden after H1N1 influenza vaccination. *Neurology*.

[191] Wijnans L., Lecomte C., de Vries C. (2013). The incidence of narcolepsy in Europe: before, during, and after the influenza A(H1N1)pdm09 pandemic and vaccination campaigns. *Vaccine*.

[192] Li H., Ye R., Pei L., Ren A., Zheng X., Liu J. (2013). Caesarean delivery, caesarean delivery on maternal request and childhood overweight: a Chinese birth cohort study of 181–380 children. *Pediatric Obesity*.

[193] Jense R. G., Koch A., Homøe P., Bjerregaard (2013). Tobacco smoke increases the risk of otitis media among Greenlandic Inuit children while exposure to organochlorines remain insignificant. *Environment International*.

[194] Csákányi Z., Czinner A., Spangler J., Rogers T., Katona G. (2012). Relationship of environmental tobacco smoke to otitis media (OM) in children. *International Journal of Pediatric Otorhinolaryngology*.

[195] Erdivanli O. C., Coskun Z. O., Kazikdas K. C., Demirci M. (2012). Prevalence of otitis media with effusion among Primary School Children in Eastern Black Sea, in Turkey and the effect of smoking in the development of otitis media with effusion. *Indian Journal of Otolaryngology and Head and Neck Surgery*.

[196] Yuan Y. H., Sun J. D., Wu M. M., Hu J. F., Peng S. Y., Chen N. H. (2013). Rotenone could activate microglia through NF*κ*B associated pathway. *Neurochemical Research*.

[197] Pereira G., Haggar F., Shand A. W., Bower C., Cook A., Nassar N. (2013). Association between pre-eclampsia and locally derived traffic-related air pollution: A Retrospective Cohort Study. *Journal of Epidemiology and Community Health*.

[198] Ozden M. G., Tekin N. S., Gürer M. A. (2011). Environmental risk factors in pediatric psoriasis: A Multicenter Case-Control Study. *Pediatric Dermatology*.

[199] Glynn A., Thuvander A., Aune M. (2008). Immune cell counts and risks of respiratory infections among infants exposed pre- and postnatally to organochlorine compounds: A Prospective Study. *Environmental Health: A Global Access Science Source*.

[200] Stølevik S. B., Nygaard U. C., Namork E. (2011). Prenatal exposure to polychlorinated biphenyls and dioxins is associated with increased risk of wheeze and infections in infants. *Food and Chemical Toxicology*.

[201] Jaakkola J. J. K., Gissler M. (2005). Maternal smoking in pregnancy as a determinant of rheumatoid arthritis and other inflammatory polyarthropathies during the first 7 years of life. *International Journal of Epidemiology*.

[202] Burt M. A., Tse Y. C., Boksa P., Wong T. P. (2013). Prenatal immune activation interacts with stress and corticosterone exposure later in life to modulate N-methyl-d-aspartate receptor synaptic function and plasticity. *International Journal of Neuropsychopharmacology*.

[203] Yoshimi N., Futamura F., Hashimoto K. (2013). Prenatal immune activation and subsequent peripubertal stress as a new model of schizophrenia. *Expert Reviews in Neurotherapy*.

[204] Patelarou E., Girvalaki C., Brokalaki H., Patelarou A., Androulaki Z., Vardavas C. (2012). Current evidence on the associations of breastfeeding, infant formula, and cow's milk introduction with type 1 diabetes mellitus: a systematic review. *Nutrition Reviews*.

[205] López-Serrano P., Pérez-Calle J. L., Pérez-Fernández M. T., Fernández-Font J. M., Boixeda de Miguel D., Fernández-Rodríguez C. M. (2010). Environmental risk factors in inflammatory bowel diseases. Investigating the hygiene hypothesis: A Spanish Case-Control Study. *Scandinavian Journal Gastroenterology*.

